# DAPIT Over-Expression Modulates Glucose Metabolism and Cell Behaviour in HEK293T Cells

**DOI:** 10.1371/journal.pone.0131990

**Published:** 2015-07-10

**Authors:** Heidi Kontro, Giuseppe Cannino, Pierre Rustin, Eric Dufour, Heikki Kainulainen

**Affiliations:** 1 Tampere Centre for Child Health Research, University of Tampere, Tampere, Finland; 2 Institute of Biomedical Technology, University of Tampere, Tampere, Finland; 3 INSERM UMR 1141, Paris, France; 4 Université Paris 7, Paris, France; 5 Department of Biology of Physical Activity, University of Jyväskylä, Jyväskylä, Finland; University of Padova, ITALY

## Abstract

**Introduction:**

Diabetes Associated Protein in Insulin-sensitive Tissues (DAPIT) is a subunit of mitochondrial ATP synthase and has also been found to associate with the vacuolar H^+^-ATPase. Its expression is particularly high in cells with elevated aerobic metabolism and in epithelial cells that actively transport nutrients and ions. Deletion of DAPIT is known to induce loss of mitochondrial ATP synthase but the effects of its over-expression are obscure.

**Results:**

In order to study the consequences of high expression of DAPIT, we constructed a transgenic cell line that constitutively expressed DAPIT in human embryonal kidney cells, HEK293T. Enhanced DAPIT expression decreased mtDNA content and mitochondrial mass, and saturated respiratory chain by decreasing H^+^-ATP synthase activity. DAPIT over-expression also increased mitochondrial membrane potential and superoxide level, and translocated the transcription factors hypoxia inducible factor 1α (Hif1α) and β-catenin to the nucleus. Accordingly, cells over-expressing DAPIT used more glucose and generated a larger amount of lactate compared to control cells. Interestingly, these changes were associated with an epithelial to mesenchymal (EMT)-like transition by changing E-cadherin to N-cadherin and up-regulating several key junction/adhesion proteins. At physiological level, DAPIT over-expression slowed down cell growth by G1 arrest and migration, and enhanced cell detachment. Several cancers also showed an increase in genomic copy number of *Usmg5* (gene encoding DAPIT), thereby providing strong correlative evidence for DAPIT possibly having oncogenic function in cancers.

**Conclusions:**

DAPIT over-expression thus appears to modulate mitochondrial functions and alter cellular regulations, promote anaerobic metabolism and induce EMT-like transition. We propose that DAPIT over-expression couples the changes in mitochondrial metabolism to physiological and pathophysiological regulations, and suggest it could play a critical role in H^+^-ATP synthase dysfunctions.

## Introduction

DAPIT is a 58 amino acid peptide first discovered in insulin-sensitive tissues of the streptozotocin-diabetic rat model [1]. It is a component of the F_o_ subunit of the mitochondrial H^+^-ATP synthase (F-ATPase) [2–4] and its knock-down results in the loss of this enzyme [5]. Recently we found that DAPIT is also a component of the vacuolar proton pump (V-ATPase) [6].

The gene encoding DAPIT is *Usmg5* that is well conserved from insects to vertebrates underlining its potentially important function. A histological analysis of DAPIT in rat and human tissues revealed an elevated expression in cells with a high aerobic metabolism and in epithelial cells involved in the active transport of nutrients and ions [6].

Interestingly, DAPIT expression appears to be modulated in various disease models. Streptozotocin (STZ) induction of diabetes in rats caused a down-regulation of DAPIT mRNA in insulin-sensitive tissues [1], but it increased DAPIT protein levels, suggesting post-transcriptional regulation [6]. In diabetic neuropathies, hyperglycaemia up-regulates the DAPIT protein in the Schwann cells of neonatal rats [7]. DAPIT is also enriched in the brain synaptosomes of a murine model of Parkinson’s disease [8]. In addition, Gene Expression Omnibus [GEO] database [9] screening suggests that the *Usmg5* transcript is up-regulated in various cancers (GEO accession GDS1792 [10], GDS3330 [11], GDS3754 [12], GDS2755 [13]), in adipose tissue of high weight gainers (GDS 2319 [14]) and in cardiac deficiencies (GDS487, GDS696); but, since post-trancriptional regulations seem to play an important role in DAPIT synthesis, it is difficult to estimate the consequences this upregulation could have at the functional level.

As a component of the H^+^-ATP synthase, DAPIT is involved in mitochondrial oxidative phosphorylation (OXPHOS), which is the major source of ATP in aerobic organisms. In various diseases, including cancer, diabetes, cardiopathies and degenerative diseases, metabolic stress lead to changes in OXPHOS activity and properties, altering mitochondrial parameters such as respiration, membrane potential, ATP production, ROS generation and mitochondrial mass. Such changes can be either beneficial (partly complementing the defects caused by the disease) or detrimental (precipitating its pathological consequences). In addition, changes in OXPHOS activity are known to elicit retrograde regulations, further altering the cellular metabolism. For example, tumour cells shift from oxidative ATP generation to glycolytic production of energy, even under normoxic conditions (the so-called Warburg effect) [15,16]. A key regulator of this effect is the nuclear stabilization of hypoxia-inducible factor 1α (Hif1α). Hif1 signalling up-regulates glycolysis and controls mitochondrial function, cell proliferation and angiogenesis while repressing apoptosis [15,17]. Hif1α activation usually requires hypoxia, but it is also observed in normoxic conditions in response to increased mitochondrial ROS production and/or accumulation on tricarboxylic acid cycle (TCA) intermediates [18,19]. Changes in respiratory chain function can also be sensed by mitochondrial sirtuins (Sirt 3–5) that modulate the activity of metabolic enzymes via protein deacylation or mono-ADP-ribosylation [20]. In particular, Sirt3, a NAD^+^-dependent deacetylase is able to activate many protein targets, including respiratory chain complex I, acetyl-CoA synthetase 2 and glutamate dehydrogenase, leading to enhanced function of TCA and increased respiration [21,22].

As *Usmg5* mRNA or DAPIT is up-regulated in various diseases and metabolic disorders known to be associated with mitochondrial functions, we aimed to study the effects of DAPIT over-expression at the cellular level. DAPIT was stably transfected into human embryonic kidney cells, HEK293T, and we studied cell morphology-, mitochondria-, nuclei-, cell junction-, behaviour- and metabolism-related parameters. We show that DAPIT over-expression modulates mitochondrial activity causing a cellular regulation that promote glycolysis and induce EMT-like transition.

## Materials and Methods

### Plasmid DNA constructs

The full-length DAPIT cDNA was originally cloned in pCR-TOPO vector (Invitrogen, Carlsbad, CA, USA) [1]. The DAPIT coding sequence–including ten nucleotides from the 5’ NCR–was recloned by PCR with the pEGFP sequence of the pIRES2-EGFP vectors (Clontech Laboratories, Palo Alto, CA, USA). The primers used were 5’- acgaattcgattgaagtcatggctggccca –3’ and 5’- tcgggatccttatgttgctttcacagctggggt –3’. The PCR reactions consisted of cycles at 96°C for 2 min, 4x (96°C for 30 s, 50°C for 1 min, 72°C for 30 s), 25x (96°C for 30 s, 60°C for 1 min, 72°C for 30 s) and 72°C for 10 min. The DAPIT amplicon was purified, cloned into the pIRES2-EGFP vector with *Eco*RI and *Bam*HI restriction enzymes (MBI Fermentas GmbH, Leon-Rot, Germany; ClontechLaboratories, Palo Alto, CA, USA) and amplified in One Shot TOP 10 bacteria (Invitrogen). The insert size (~204 bp) was confirmed by restriction enzyme digestion, and the insert DNA was fully sequenced. The construct was used for stable transfection of HEK293T cells.

### Cell culture, transfections and RT-PCR

HEK293T cells (ATCC, crl-3216) were cultured in Dulbecco's modified Eagle's medium (Sigma-Aldrich, Ayshire, UK or Gibco BRL, Paisley, Scotland, UK), containing 4.5 g/l of D-glucose, 10% foetal calf serum (Sigma), 50 μg/ml uridine, 1 mM sodium pyruvate, 2 mM L-glutamine, 100 U penicillin and 100 μg/ml of streptomycin (Gibco BRL) at 37°C in an incubator with 5% CO_2_. Transfections were performed using Lipofectamine according to the manufacturer's protocol (Invitrogen). Transfection efficiency was estimated by flow cytometry using GFP fluorescence. Twenty-five Geneticin-resistant clones (Calbiochem/Merck KGaA, Darmstadt, Germany; 2 mg/ml) were selected and combined to form the polyclonal cell line. Total RNAs from pIRES2-EGFP and DAPIT-pIRES2-EGFP stably transfected cells were extracted by RNeasy Mini Kit (Qiagen), and 1 μg total RNA was used for RT-PCR using M-MuLV reverse transcriptase, as suggested by the provider (MBI Fermentas). The obtained cDNAs of control and transgenic cells were multiplied by PCR as indicated above.

### Fluorescence microscopy

For live imaging of mitochondria and lysosomes, the cells grown on poly-L-lysine coated cover slips (Sigma) were washed with PBS and incubated in a medium containing 100 nM Mitotracker Red (Invitrogen) or 100 nM Lysotracker red (Invitrogen) for 10–30 min at 37°C. Mitotracker-stained cells were PBS washed and maintained in DMEM medium for 1 hour at 37°C before observation. For immunofluorescence microscopy, cells fixed in 4% paraformaldehyde (Sigma) for 15 min were permeabilized with 0.5% Triton X-100 (MP Biomedicals,Illkirch, France) in TBS (10mM Tris, 0.9% NaCl, pH 8.0 (Sigma)) for 10 min. Non-specific epitopes were blocked by using 5% w/v non-fat milk powder, 2% w/v BSA (Sigma) for 30 min. Samples were incubated in TBS-T (TBS with 0.1% Tween (Sigma)) with the primary antibody (αD15C, 1:300) [5] for 2 h at room temperature, washed for 3x5min and incubated in Alexa Fluor 488 or 568 goat anti-rabbit, Alexa Fluor 568 goat anti-mouse or Alexa Fluor 549 chicken anti-mouse IgG secondary antibodies (Molecular Probes, Eugene, Oregon, USA, 1:4000) for 1 hour. Coverslips were mounted on slides using Vectashield mounting medium (Vector Laboratories, Burlingame, CA, USA), and samples were examined by confocal microscopy at 100x magnification using a Perkin Elmer-Cetus/Wallac UltraView LCI system (Wellesley, MA, USA) equipped with appropriate excitation and emission filters, an Andor iXon DV885 EMCCD camera and the Andor iQ software (Andor, Belfast, UK), or with a conventional fluorescence microscope at 40x and 60x magnification (Olympus BX60, Olympus Corporation, Japan). Images were further processed using Corel Photo-Paint 11 (Corel Corporation).

### Mitochondrial copy number calculation and citrate synthase activity

For mtDNA copy-number analysis, total DNA was prepared as reported in Fukuoh et. al. [23]. The isolated DNA from 0.4X10^6^ cells were resuspended in TE buffer (pH 8.0), purified and quantified by Nanodrop. Relative mtDNA copy number was measured by real-time qPCR using primers for mitochondrial COXII subunit (Forward cgtctgaactatcctgcccg, Reverse tggtaagggagggatcgttg) and nuclear APP (Forward tttttgtgtgctctcccaggtct, Reverse tggtcactggtttggc) in a StepOnePlus instrument (Applied Biosystems>place) using Fast SYBR Green Master Mix (Applied Biosystems) under the manufacturer’s recommended conditions, with 20 sec of enzyme activation at 95°C, followed by 40 cycles of 95°C for 3 sec and 60°C for 30 sec.

The activity of citrate synthase of cells was measured using a kit (Sigma-Aldrich, CS0720) according to the manufacturer´s instructions with an automated KoneLab device (Thermo Scientific, Vantaa, Finland).

### Oxygen consumption and fluorescence biomarkers

The mitochondrial measurements in living cells were performed as in Cannino et. al. [24]. Oxygen consumption was measured with a Clark-type electrode (Oxygraph, Hansatech Instruments Ltd, Norfolk, UK). Intact cell respiration was recorded from 1x10^7^ cells suspended in 500 μl of DMEM medium at 37°C. Maximum respiration was obtained by FCCP titration (5–9 μM). Oxygen consumption was stopped with 150 nM rotenone, 30 ng/ml antimycin A, 100 μM Cyanide or 100–200 nM Oligomycin (Sigma). Oxygen consumption from 1x10^7^ cells permeabilized by 80 μg/ml digitonin was recorded in respiratory buffer A (225 mM sucrose, 75 mM mannitol, 10 mM Tris-buffer pH 7.4, 10 mM KCl, 10 mM KH_2_PO_4_, 5 mM MgCl2, 1mg/ml BSA (Sigma)) at 37°C. The substrate concentrations were 10 mM ADP, 5 mM pyruvate + 5 mM malate for complex I, 10 mM succinate for complex II, and 50 μM TMPD and 1 mM ascorbate for complex IV. All measurements were corrected by subtracting the residual oxygen consumption present after full inhibition of the respiratory chain.

For the mitochondrial mass, membrane potential and superoxide measurements, flow cytometry assays were used. In the absence of G418, 4x10^5^ (Vector) and 4.5x10^5^ (DAPIT) cells were seeded in culture medium. After overnight culture, the subconfluent cells were treated with 200 nM 10-nonyl acridine orange (NAO; Invitrogen,) for 30 min at 37°C, 200nM tetramethyl rhodamine methyl ester (TMRM; Invitrogen,), for 30 min at 37°C or 2.5 μM MitoSox (Invitrogen,), for 45 min at 37°C. The staining was stopped by replacing the medium with 1xPBS, and cells were kept at 37°C (NAO and TMRM) or on ice (MitoSox) until measured. Negative controls for mitochondrial membrane potential were obtained by adding 10 μM FCCP before flow cytometry analysis.

The fluorescence was counted from 40,000 cells using a BD Accuri C6 flow cytometry (BD Biosciences). The region of interest was defined by using the forward scatter/side scatter values, excluding the debris and dead cells. The staining was measured either by using 488 nm (bandpass) excitation and emission of FL2 (585 ± 40 nm) for NAO and TMRM, FL3 (620 ± 15 nm) for Mitosox and FL1 (533 ± 40 nm) for GFP. The fluorescence compensations were estimated independently for each series of experiments. All measurements provided as “relative to mitochondrial (mt) content” were normalized by NAO quantification, whereas measurements provided as “*per* cell” were normalized to the cell count.

### Isolation of mitochondria and complex V activity

For crude extraction of mitochondria, the cells from four 17.5 cm^2^ or 8–10 10 cm^2^ culture plates were collected by centrifugation at 250 *g* for 3 min at room temperature. The rest of the protocol was carried out in a cold room (+4 C) on ice. The cells were bloated in 5.5 ml of hypotonic buffer (10mM NaCl, 1.5mM MgCl2, 10mM Tris-HCl pH 7.5 (Sigma), protease inhibitor coctail (Roche, Mannheim, Germany)) for 8–13 min, ruptured with eight strokes of teflon pestle. 4 ml of 2.5X MS buffer (700 mM sucrose, 2.5 mM EDTA, 12.5 mM Tris-HCl pH 7.5, protease inhibitors) was added. To remove nuclei and cell debris, the samples were centrifuged at 1,300 *g* for 10 min. Mitochondria from the supernatant was pelleted by centrifugation at 17,000 *g* for 15 min, and diluted to 0.5–1 ml of 1X MS buffer (0.28 mM sucrose, 5 mM Tris-HCl, 1 mM EDTA pH 7.5, protease inhibitors).

Fifteen ml of 1.5 M and 1.0 M sucrose in buffer (10mM Tris-HCl pH 7.4, 1 mM EGTA, 0,1% BSA, protease inhibitors) were layered in ultracentrifuge tubes. The crude extract of mitochondria was added on the top of sucrose layers and centrifuged at 60 000 *g* for 20 min at 4°C. The resulting fraction of mitochondria in the interphase of sucrose layers was collected, the volume measured and slowly (15–20 min) diluted on ice for 4X with 0.2M mannitol in Tris-EGTA-BSA buffer. Finally, the mitochondria were pelleted at 17,000 *g* for 15 min at 4°C, diluted to 40–50 μl of 1X MS buffer and stored at -80°C.

The oligomycin-sensitive complex V activity was spectrophotometrically measured in the backward direction using lactic dehydrogenase and pyruvate kinase as coupling enzymes, as essentially described elsewhere [25,26].

### Cell growth, mortality, and synchronization

Fifteen thousand cells were seeded on a 24-well culture plate (Nunclon, Thermo Scientific) in 500 μl of culture medium in the presence of antibiotics (Penicillin-Streptomycin & G418). Cell proliferation and mortality were followed for five days by counting the living and dead cells in a Burker hemocytometer after trypan blue labelling (0.4%; Sigma).

The control and DAPIT overexpressing cells were synchronized by a double thymidine block method (DIAMONDS Deliverable 1-D1.1.3, ResearchGate.net) for more accurate follow up of cell division. At 30% confluence in 24-well culture plate, the cells were washed twice with 1x PBS and 1 ml of cell culture medium supplemented with 2 mM thymidine (Sigma) was added for 18 hours. Thymidine was washed out and the cell divisions were released by adding fresh cell culture medium for nine hours. This was followed by another thymidine step for 17 hours after which the cells progress synchronously through G2- and mitotic phase. Upon the release from the thymidine block, the cells were cultured in normal medium for 4, 8, 12, 16 and 24 hours in order to follow the cell cycle progress. At each time point the cells were collected, pelleted, stained with 250 μl of PI staining solution (25μg/ml propidium iodide, 100μg/ml RNAse A, 0.1% sodium citrate, 0.1% Triton X-100 (Sigma)) for 20 min on ice and measured by flow cytometry (488 nm excitation, >670 nm emission; FL3). The number of cells (arbitrary units) was blotted against the DNA content at each time point. The test was repeated for four times.

### Migration and adhesion

The migration was studied by scratch wound test in a 12-well culture plate (Nunclon). The cells were grown confluent, and fresh medium was provided three hours prior to starting the test. After 1 hour-treatment with 20 μg/ml Mitomycin C (Sigma) cells were scratched with a tip, washed and incubated overnight at 37°C. After fixation with 4% PFA for 15 min and PBS washing, cells were stained with crystal violet (0.5 mM) for 5 min in 70% ethanol. The cells migrating in the scratched area were counted using a phase contrast microscope (Axiovert 200 M, Zeiss). Cell attachment was studied using PMS/MTS (Promega, Madison, WI, USA) according to the manufacturers’s protocol. Detachment was determined according to the protocol used in migration assays in a 48-well culture plate (Nunclon). The empty areas of detached cells in the bottom of wells were quantified by ImageJ (imagej.nih.gov/ij) analysis.

### Western blot analysis

Proteins from subconfluent (approximately 50–70%) cells were extracted in PBS containing 1% Triton X100 and protease, followed by incubation on ice for 30 min and centrifugation at 12,000 *g* for 1 minute. The protocol applied by Teittinen et al. [27] was followed to obtain nuclear extracts. Briefly, subconfluent cells were washed with PBS and collected by centrifugation. The cells were resuspended in 5 ml hypotonic buffer (10 mM Hepes (pH 7.9), 10 mM KCl, 1.5 mM MgCl_2_, 0.5 mM DTT (Sigma)) and broken on ice using a Dounce homogenizer. Nuclei were pelletted by centrifugation (228 *g*, 5 min, +4°C) and purified by isopycnic centrifugation (1,430 *g*, 5 min, +4°C) on a two-step sucrose gradient: 250 mM sucrose, 10 mM MgCl_2_ *vs* 880 mM sucrose, 0.5 mM MgCl_2_. The proteins of this nuclear fraction were extracted as described above.

The protein concentration was determined by the Bradford method [28]. Then, 20 μg of cellular and 50 μg of nuclear protein were used for SDS-PAGE analysis according to Laemmli et al. [29] and transferred to Hybond-C extra nitrocellulose membrane (Amersham plc, Buckinghamshire, UK). Non-specific epitopes were masked, exposing membranes to 5% freeze-dried fat-free milk in TBS-T for 1 hour. Primary antibodies (see Table 1) were incubated for 2 hours. After washings, the blots were incubated with the secondary antibody: peroxidase-conjugated swine anti-rabbit and rabbit anti-mouse (DAKO, Clostrup, Denmark) 1:2,000, or Peroxidase Horse Anti-Goat IgG (H+L; Vector Laboratories) 1:10,000 for 1 hour. Subsequently, the blots were washed, and the signal was detected by enhanced chemiluminescent ECL reagent (Amersham) according to the manufacturer’s protocol. The blots were visualized on Super RX medical X-ray film (Fujifilm Corporation, Tokyo, Japan) and the bands quantitated by Kodak imaging software (Eastman Kodak Company, US). The protein expression was normalized to the house-keeping protein gamma-tubulin, and additionally to mitochondrial content (NAO result) in the case of the mitochondrial proteins.

**Table 1:** List of primary antibodies and dilutions used in Western blot.

Antibody	Dilution	Host	Manufacturer
ATP5a	1:4000	Mouse monoclonal	Abcam, #MS502
β-actin	1:5000	Mouse monoclonal	Sigma, #A5316
β-catenin	1:400	Mouse monoclonal	Transduction Laboratories, BD Biosciences, #610153
Connexin 43	1:1000	Rabbit polyclonal	Sigma, #C6219
αD15C (anti-DAPIT)	1:160	Rabbit polygonal	Custom made [5]
E-cadherin	1:1000	Rabbit polyclonal	Santa Cruz, #sc-7870
γ-tubulin	1:4000	Mouse monoclonal	Sigma, #T5326
GFP	1:10000	Mouse monoclonal	Zymed, #33–2600
Hif1α	1:1000	Mouse monoclonal	Abcam, #10625, ab8366
Histone H1	1:500	Mouse monoclonal	Santa Cruz, #sc-8030
HSP60	1:600	Mouse monoclonal	Sigma, #4149
Integrin α2	1:200	Mouse monoclonal	Santa Cruz, #sc-13546
N-cadherin	1:1000	Mouse monoclonal	Sigma, #C2542
NDUFS3	1:10000	Mouse monoclonal	Abcam, # 14711
PGC1α	1:3000	Rabbit polyclonal	Millipore, #516557
RhoA	1:200	Mouse monoclonal	Santa Cruz, #sc-418
Sirt3	1:400	Goat polyclonal	Abcam, #118334
Smooth muscle actin	1:200	Mouse monoclonal	Sigma, #A5228
VDAC1/Porin	1:1000	Mouse monoclonal	Nordic BioSite, #MSA03
Vimentin	1:200	Goat polyclonal	Millipore, #AB1620
Zo-1	1:300	Mouse monoclonal	Invitrogen, #339100

### Glucose and lactate test

The glucose consumption and lactate production were measured from culture media of the cell proliferation test and results were normalized with concomitant cell number. Glucose and lactate levels were analysed using the enzymatic-amperometric method and chip-sensor technology (Biosen C-line Sport, EKF Diagnostic, Magdeburg, Germany).

### Oncomine data analysis

We used the Oncomine Cancer Genomics Data Analysis tool [30] to mine *Usmg5* copy number profiles in a large subset of carcinomas and cancer cell lines [31–48]. In the dataset information of cancers both significant differences (p≤0.05) and fold changes (≥ 1) were reported. The number of DNA copies (= 2*(2^y-axis value)) were calculated as advised in Oncomine instructions.

### Statistical analysis

Comparisons between cell lines were performed by using Mann-Whitney U test.

## Results

For the sake of simplicity, from now on we will call “control cells” the cells transfected with empty pIRES2-EGFP vector and “DAPIT cells” the ones over-expressing transgenic DAPIT. The transgene is co-transcribed with a cytosolic GFP reporter, independent from fusion protein.

### Mitochondrial mass, mtDNA and DAPIT over-expressing cells

**Fig 1:**
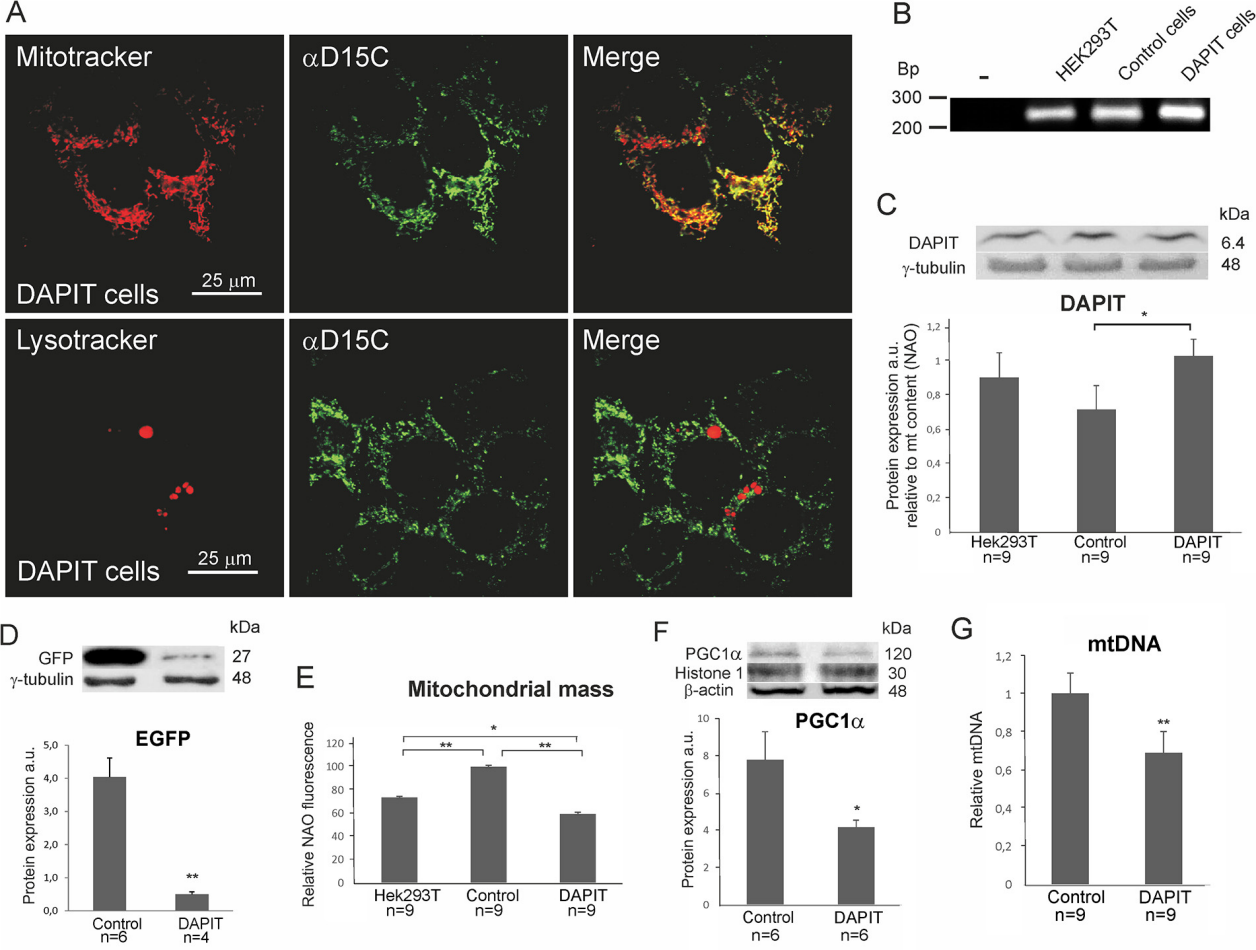
DAPIT over-expression decreased mitochondrial mass and mtDNA content in DAPIT cells. (A) Representative confocal microscopy images of cells stained by Mitotracker and Lysotracker (100 nM, 10–30’, 37°C) and anti-DAPIT antibody, αD15C. (B) mRNA expression of the *Usmg5* by semi-quantitative RT-PCR. Protein levels estimated by Western blot of (C) DAPIT and (D) EGFP. (E) Mitochondrial mass was measured by flow cytometry of NAO stained cells (200nM, 30’, 37°C). (F) Protein level of PGC1α from nuclear extract. (G) mtDNA content by quantitative PCR. The error bars are S.D. and asterisks indicate: *p<0.05, **p<0.01.

As DAPIT was reported a Fo subunit of H^+^-ATP synthase, the immunofluorescence analysis of DAPIT cells showed clear co-localization of mitochondrial and DAPIT signals (Fig 1A). Importantly, we observed very few DAPIT-positive lysosomes. Knowing that mitochondria that are targeted for degradation (*e*.*g*. through mitophagy) would lead to a transient localization of DAPIT into lysotracker positive compartments, our results suggest a pure mitochondrial location of this protein.

In this study, we emphasize the comparison of DAPIT cells to control ones due to vector transduction and following culture conditions. For assuring our cell model, the mRNA and protein level and mitochondrial mass from HEK293T cells are also reported. As mitochondrial mass is sensitive to H^+^-ATP synthase impairments [23,49,50], the concomitant differences in mass between cell lines are normalized into reported mitochondrial parameters.

The expression of transgene was controlled by reverse transcriptase-PCR (RT-PCR) and Western blot analysis. DAPIT cells presented higher level of *Usmg5* messenger RNA compared to the HEK293T and control cells (Fig 1B) demonstrating the functionality of the DAPIT construct.

The expression of DAPIT protein in the three cell lines is shown in Fig 1C. DAPIT expression was slightly decreased in control cells (vehicle) as compared to HEK293T cells. A mild but not significant increase was seen between HEK293T and DAPIT cells, but significantly higher expression was observed in DAPIT than in control cells (p<0.05). The green fluorescent protein expression appeared lower in DAPIT cells (Fig 1D) indicating that translation from the 5’ RNA CAP is more efficient than internal ribosome entry, as previously reported [51].

In order to study the effect of DAPIT over-expression on mitochondrial physiology, we measured mitochondrial mass using the mitochondria specific dye NAO in HEK293T, control and DAPIT cell lines (Fig 1E). Mitochondrial mass in DAPIT cells was significantly lower than in control and HEK293T cells, while it was intermediate in HEK293T cells.

To verify the mitochondrial mass in control and DAPIT cells, we measured mtDNA copy number and nuclear level of mitochondrial biogenesis regulating transcription factor PGC1α. In line with NAO results, both the translocation of PGC1α to the nuclei and the mtDNA content were decreased in DAPIT cells (Fig 1F and 1G). These results indicate that mitogenesis is decreased in DAPIT cells.

### Metabolic activity of mitochondria

**Fig 2:**
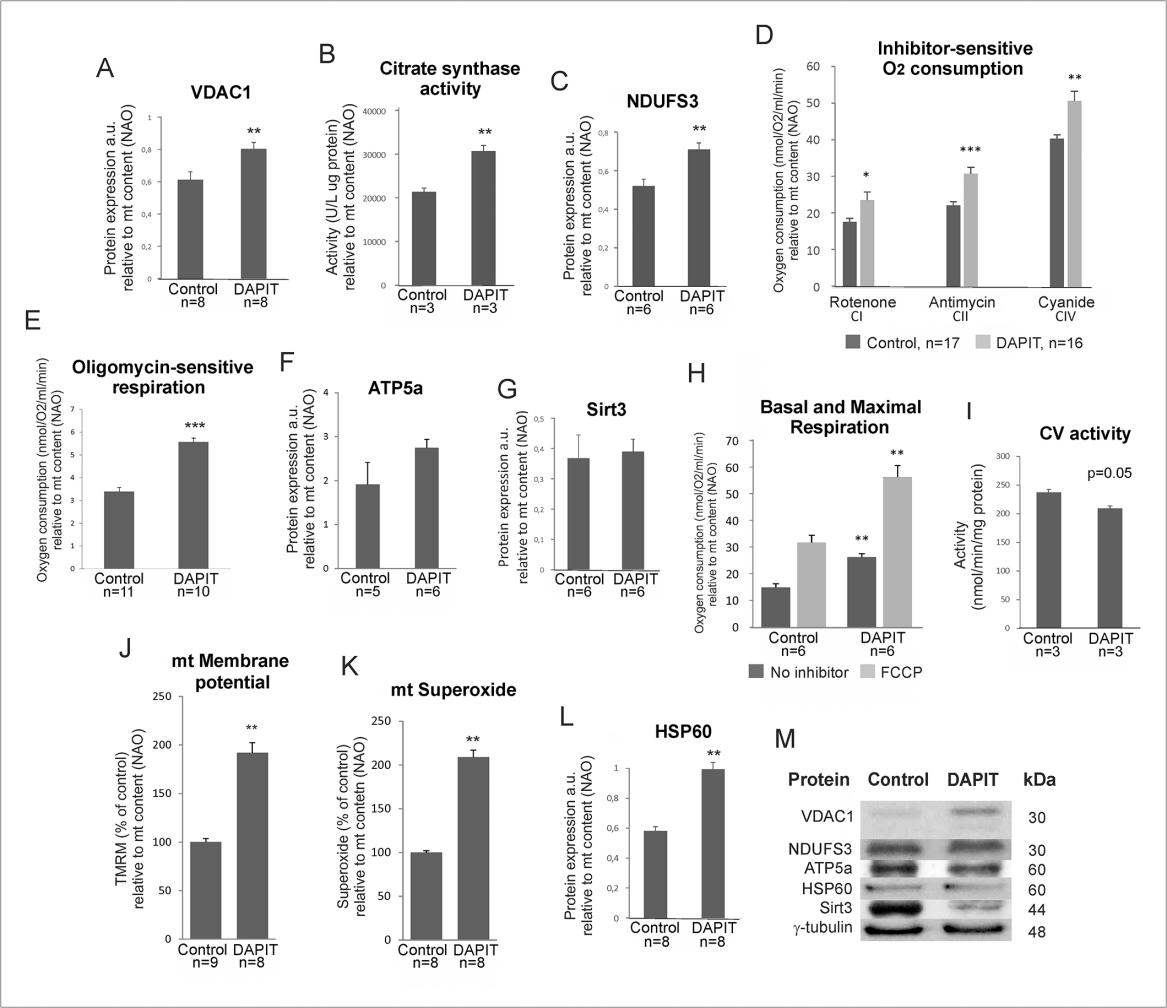
Mitochondrial activity in DAPIT over-expression (relative to mitochondrial mass (NAO)). (A) Protein level of VDAC1 by Western blot of cellular lysates. (B) Citrate synthase activity was measured by spectrophotometric analysis from protein extracts of control and DAPIT cells. (C) Protein level of NDUFS3. (D) Inhibitor-sensitive oxygen consumption of complexes I, II, IV and (E) complex V in digitonin-permeabilized and intact cells. Protein level of (F) ATP5a and (G) Sirt3. (H) Basal and maximal respiration by oxygen consumption of living cells. (I) Spectrophotometric analysis applied for measuring CV activity in backward direction using lactic dehydrogenase and pyruvate kinase as coupling enzymes. Mitochondrial (J) membrane potential and (K) superoxide levels measured by flow cytometry of TMRM (200nM, 30', 37°C) and Mitosox (2,5 μM, 45’, 37°C) stained cells. (L) Protein expression of HSP60. (M) Representative immunoblots. The error bars are S.D. and asterisks indicate: *p <0.05, **p<0.01 and ***p<0.001.

We next estimated the effect of DAPIT over-expression on mitochondrial protein transport, TCA cycle activity, respiratory chain activity in intact cells and oxygen consumption driven by complexes I, II and IV in permeabilized cells. The results are reported both at cellular level (S1 Fig) and in relation to mitochondrial content (NAO normalized results, Fig 2).

The expression of VDAC1 (Fig 2A and 2M) and the activity of citrate synthase (Fig 2B) were increased significantly upon DAPIT over-expression (p = 0.001 and 0.05, respectively). These results could indicate increased transport or reduced turn-over of cytosolic substrates suitable for enhanced oxidation by TCA cycle, thereby facilitating the demands of respiratory chain.

The protein expression of C1 subunit NDUFS3 (p = 0.004) was increased in DAPIT cells (Fig 2C and 2M). In addition, both the oxygen consumption from complexes I, II and IV in digitonin-permeabilized cells and respiration of intact cells (Fig 2D and 2E) were significantly increased (p = 0.044, 0.000, 0.002, 0.000), respectively. However, the expression of F_1_ complex subunit ATP5a was unaltered (Fig 2F and 2M) suggesting that enhanced oxygen consumption seen in DAPIT cells is not due to increase in CV content. Instead, the changes in respiratory chain function are supported by unchanged expression of Sirt3 (Fig 2G and 2M), a modulator of the activity of metabolic enzymes. In addition, DAPIT cells exhibit increased basal and maximal respiration (p = 0.003 and 0.004, respectively) (Fig 2H) and decreased activity of ATP synthase (p = 0.005)(Fig 2I). These results suggest enhanced substrate availability and increased activity of TCA cycle, efficient respiration, active coupling but decreased H^+^-ATP synthase activity in DAPIT cells. Accordingly, the membrane potential (p = 0.001) and superoxide (p = 0.001) levels were increased (Fig 2J and 2K). When assessed at cell level (S1 Fig), respiration and TCA cycle activity remained unchanged between cell lines but VDAC1 expression, CV activity, membrane potential and superoxide showed the same changes as when normalized to mitochondrial mass. Taken together, these results suggest the saturation of respiratory chain due to DAPIT over-expression. The protein level of HSP60 increased significantly (p = 0.009) in DAPIT cells (Fig 2L and 2M), indicative of an appropriate maintenance of the mitochondrial proteins.

### Nuclear proteins

**Fig 3:**
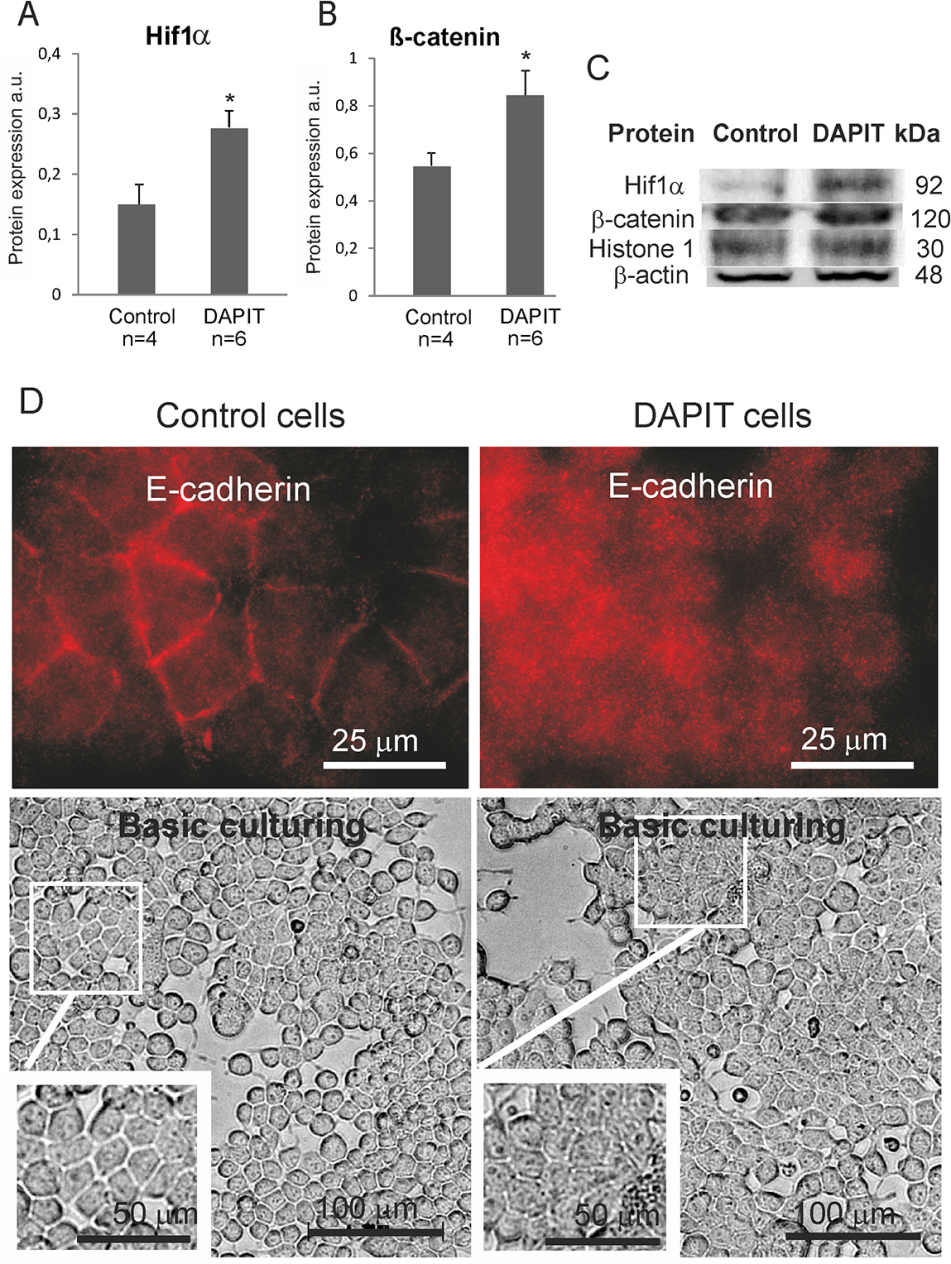
DAPIT over-expression regulates nuclear translocation of Hif1α and β-catenin leading to morphological changes. Nuclear protein level of (A) Hif1α and (B) β-catenin by Western blot. (C) Representative immunoblots. (D) Immunofluorescence of E-cadherin (upper panel, 100x magnification) and microscope view of living control and DAPIT cells (lower panel, 20x magnification). The error bars are S.D. and asterisk indicates *p <0.05.

We observed increased nuclear translocation of the Hif1α transcription factor in DAPIT cells (Fig 3A and 3C). We also observed an increased protein expression of nuclear β-catenin (Fig 3B and 3C) and relocation of E-cadherin from cell junctions to the cytosol in DAPIT cells (Fig 3D, upper panel). Altogether, these results suggest major remodeling of cellular functions in response to DAPIT over-expression, since Hif1α and β-catenin level are known to be involved in cellular dedifferentiation [52].

### Morphological analysis of cell junction and adhesion proteins in DAPIT over-expressing cells

The over-expression of DAPIT induced changes in cell morphology, from a regular cuboidal epithelial-like (control cells) to an irregularly sized and shaped morphology (DAPIT cells) with decreased intercellular separation, showing a polygonal, sheet-like appearance but unaffected cell projections (Fig 3D, lower panel), thereby suggesting an epithelial to mesenchymal transition (EMT).

**Fig 4:**
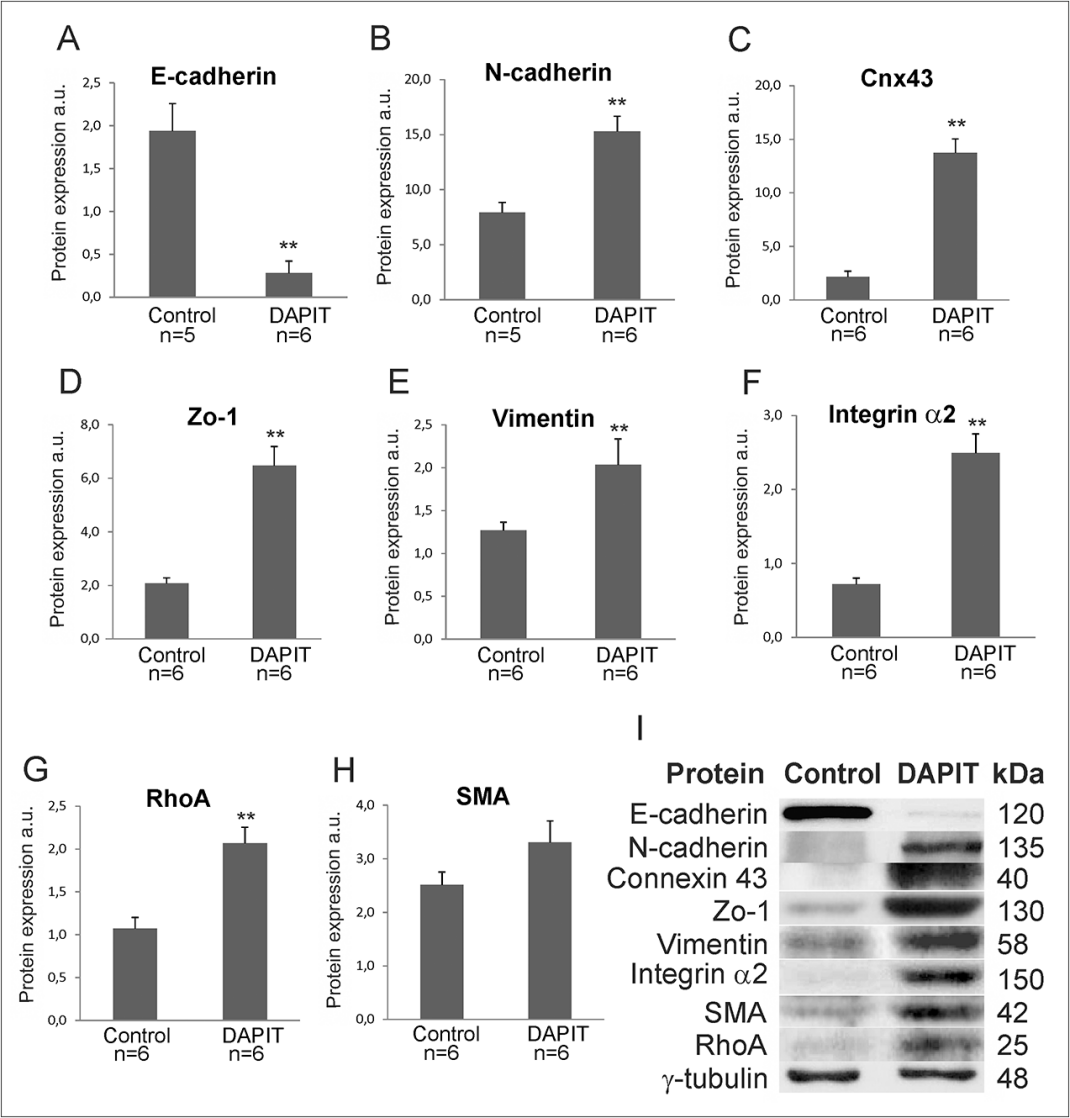
Modification in cell junction and adhesion proteins correspond to EMT-like changes in DAPIT cells. (A-H) Modulation of cell junction proteins and RhoA. Protein expression by Western blot in 20μg of cell lysate. (I) Representative immunoblots. The error bars are S.D. and asterisks indicates **p<0.01.

Due to morphological changes in DAPIT cells, we investigated the expression of several cell junction and adhesion proteins (Fig 4). Protein levels of E-cadherin decreased significantly (Fig 4A and 4I), while N-cadherin, Connexin 43, ZO-1, Vimentin, Integrin α2, and their modulator RhoA GTPase were all increased (Fig 4B–4G and 4I). We also observed increased (although non-significant) expression of the SMA (Fig 4H and 4I). Interestingly, such pattern of expression is reminiscent of the EMT observed, for example, in embryogenesis, wound healing and cancer.

### Cell growth, mortality, migration and adhesion

**Fig 5:**
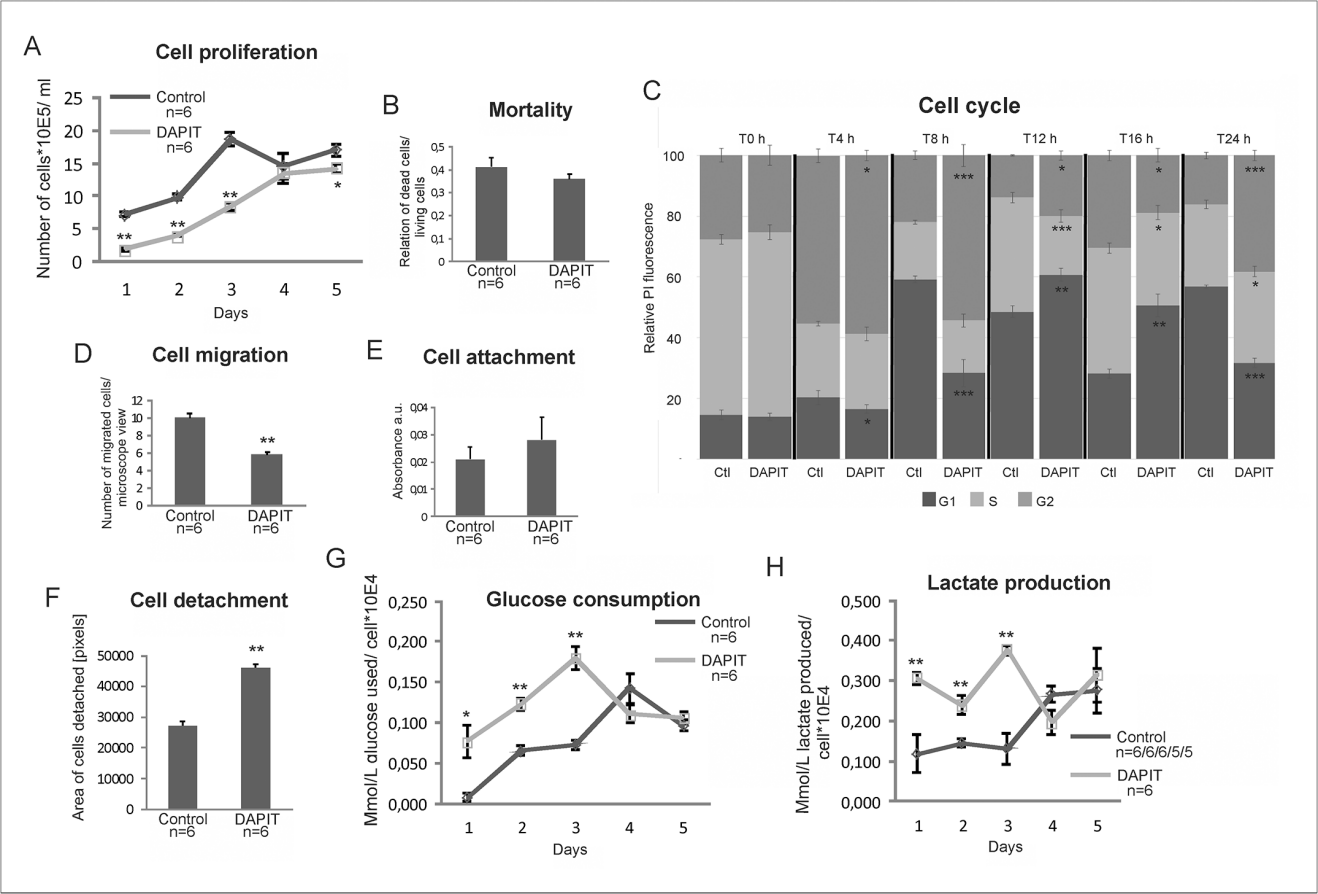
Characteristics of cell behaviour and metabolism of DAPIT over-expressing cells. (A) Cell proliferation by cell counting. (B) Mortality rate at day two of culture. (C) Flow cytometric analysis of cell cycle progression after double thymidine syncronization of the cells. (D) Cell migration by scratch wound assay. Cell adhesion capacity; (E) attachment measured by PMS/MTS test and (F) detachment according to migration test. (G) Glucose consumption and (H) lactate production measured in parallel from the culture medium of the cell proliferation test. The error bars are S.D. and asterisks indicate: *p<0.05 and **p<0.01.

In order to study the effect of DAPIT over-expression on cell physiology and to clarify the consequences of the EMT-like phenotype, we examined cell growth, mortality, cell cycle, migration and adhesion capacity (Fig 5). According to hemocytometer calculation, DAPIT cells showed slower growth during the active growing phase (Fig 5A, days 1–3). Since the mortality rate, measured at day 2, was not altered (Fig 5B), we attribute the slower cell proliferation to a reduced proliferation rather than increased cell death. To test this premise, we followed the cell cycle in synchronized cells. After withdrawal of thymidine, DAPIT cells entered the G1 phase approximately four hours later than control cells (seen at time point 4 h) (Fig 5C, dark grey). This retardation results also significant difference in S phase from 12 h on (light grey) and in G2 (medium grey) at 4 h onwards, thereby confirming the slower growth of DAPIT cells.

The unexpected decreased migration capacity (Fig 5D) of DAPIT cells is in contradiction with EMT and suggests a suppressive trait. The attachment capacity of the cells remained unchanged, whereas cell detachment was enhanced (Fig 5E and 5F), indicating adhesion characteristics typical of EMT.

### Glucose consumption and lactate production

The miss-functional ATP-synthase, reduced growth and HIF1α stabilization in DAPIT cells suggested a metabolic shift from aerobic respiration to glycolysis. Therefore, we measured, in parallel, glucose consumption and lactate production in DAPIT cells. As we anticipated, DAPIT cells consumed more glucose and produced more lactate during the exponential stage of their growth at days 1–3 (Fig 5G and 5H). Cell growth reached a plateau at day 4, and this was associated with a decreased glucose consumption and lactate production, a metabolic switch attributable to cell quiescence (see Valcourt et al. [53] for a review).

### *Usmg5* copy number in cancers

Since DAPIT over-expression induced EMT and glycolytic switch in HEK293T cells, we tested if DAPIT is over-presented in cancers. The Oncomine Cancer Genomics database revealed a duplication (4 copies) of *Usmg5* copy number in a large panel of cancers (Table 2). *Usmg5* was ranked within 10% of top genes duplicated in various brain, pancreas and liver cancers, within 15% in sarcomas, kidney, lung and gastric cancers, and within approximately 20% in leukemia, lymphoma, and breast- and ovarian cancers. These data strongly suggest a role for DAPIT over-expression in cancers.

**Table 2:** Cancers expressing increased genomic *Usmg5* copy number in Oncomine cancer genomics database.

Classification	Cancer type	DNA copy number	*Gene rank %	Oncomine datasets
Brain	Astrocytoma	4,40	1	Beroukhim Brain [31]
	Astrocytoma	4,50	3	TCGA Brain 2 [N/A]
	Astrocytoma	4,36	4	Kotliarov Brain [32]
	Astrocytoma	4,07	12	Northcott Brain 4 [33]
	Head and Neck Cancer Cell Line	4,13	2	Beroukhim Multi-cancer [34]
	Head and Neck Cancer	4,11	5	Beroukhim Multi-cancer [34]
	Head and Neck Cancer	4,13	24	Barretina CellLine 2 [35]
	Mixed Glioma	4,89	1	TCGA Brain 2 [N/A]
	Oligodendroglial Tumor	4,73	2	Kotliarov Brain [32]
	Oligodendroglial Tumor	5,07	3	TCGA Brain 2 [N/A]
	Oligodendroglial Tumor	4,29	7	Beroukhim Brain [31]
Pancreas	Pancreatic Cancer	4,19	6	Barretina CellLine 2 [35]
Liver	Liver Cancer	4,33	4	Rothenberg CellLine [38]
	Liver Cancer	4,18	7	Barretina CellLine 2 [35]
	Liver Cancer Precursor	4,10	29	Chiang Liver 2 [46]
Sarcoma	Dedifferentiated Liposarcoma	4,12	9	Barretina Sarcoma 2 [37]
	Myxoid/Round Cell Liposarcoma	4,12	15	Barretina Sarcoma 2 [37]
	Sarcoma	4,12	8	Barretina CellLine 2 [35]
	Sarcoma	4,12	12	Wooster CellLine 2 [N/A]
	Sarcoma	4,10	13	Rothenberg CellLine [38]
	Sarcoma Cell Line	4,14	24	Beroukhim Multi-cancer [34]
Kidney	Kidney Cancer	4,67	2	Neale Multi-cancer 2 [43]
	Kidney Cancer	4,20	12	Barretina CellLine 2 [35]
	Hereditary Clear Cell Renal Cell Carcinoma	4,07	26	Beroukhim Renal 2 [47]
Lung	Non-Small Cell Lung Carcinoma	4,13	10	Weiss Lung [40]
	Non-Small Cell Lung Carcinoma	4,18	12	TCGA Lung 2 [N/A]
Gastric	Gastric Adenocarcinoma	4,06	14	Deng Gastric [36]
	Gastric Mixed Adenocarcinoma vs. Normal	4,10	15	Deng Gastric [36]
	Gastrointestinal Stromal Tumor	4,40	17	Barretina Sarcoma 4 [37]
	Colon Adenocarcinoma	4,06	14	TCGA Colorectal 2 [N/A]
	Colorectal Cancer	4,13	13	Rothenberg CellLine [38]
	Colorectal Cancer	4,07	18	Barretina CellLine 2 [35]
	Colorectal Cancer	4,28	13	Jaiswal Multi-cancer [39]
Leukemia	Leukemia	4,46	5	Neale Multi-cancer 3 [43]
	Refractory Anemia with Excess Blasts-1 vs. Normal	4,13	13	Yoshida Leukemia [44]
	Chronic Myelomonocytic Leukemia-1 vs. Normal	4,12	14	Yoshida Leukemia [44]
	Leukemia Cell Line	4,17	20	Beroukhim Multi-cancer [34]
	Plasma Cell Leukemia	4,09	21	Chapman Myeloma 2 [45]
Breast	Mixed Lobular and Ductal Breast Carcinoma	4,11	7	Nikolsky Breast [41]
	Papillary Breast Carcinoma	4,08	11	TCGA Breast 2 [N/A]
	Mucinous Breast Carcinoma	4,08	20	Curtis Breast 2 [42]
	Lobular Breast Carcinoma	4,07	23	TCGA Breast 2 [N/A]
	Ductal Breast Carcinoma in Situ	4,06	24	Curtis Breast 2 [42]
Ovarian	Ovarian Cancer	4,09	16	Beroukhim Multi-cancer [34]
	Endometrial Endometrioid Adenocarcinomavs. Normal	4,10	17	TCGA Endometrium [N/A]
	Endometrial Serous Adenocarcinoma vs. Normal	4,16	29	TCGA Endometrium [N/A]
Lymphoma	Lymphoma	4,15	21	Wooster CellLine 2 [N/A]
	Lymphoma	4,08	28	Barretina CellLine 2 [35]
Other	Oral Cavity Squamous Cell Carcinoma vs. Normal	4,03	48	Peng Head-Neck 2 [48]

N/A indicating not available.

* Indicating % of top genes duplicated, within which *Usmg5* was ranked.

## Discussion

The OXPHOS system comprises five multi-subunit enzymes known as complexes I, II, III, IV and V. The electron transfer through complexes I-IV is coupled to proton translocation across the inner membrane. This results transmembrane electrochemical potential which is converted into chemical energy in the form of ATP by H^+^- ATP synthase (CV).

DAPIT has been shown to be a structural component of H^+^-ATP synthase and its deletion resulted in the loss of H^+^-ATP synthase [2–5]. As DAPIT mRNA and/or protein levels are increased in various diseases [6–8, 10–14] we hypothesized that in addition to its structural role, DAPIT could also be a regulatory component of H^+^-ATP synthase. In consequence, DAPIT up-regulation could lead to both structural changes and alteration in respiratory chain regulation. In the present study, we stably transfected DAPIT into HEK293T cells. The strategy we used permits both the transgene and an EGFP reporter to be translated from a single bicistronic mRNA without formation of a fusion protein. The DNA sequence of the DAPIT transgene appeared unaltered and the expression of the protein was confirmed. We emphasized the effect of DAPIT over-expression on mitochondrial level by normalizing the reported mitochondrial parameters with concomitant mass. Accordingly, DAPIT up-regulation did not alter the mitochondrial H^+^- ATP synthase levels in terms of the expression of ATP5a (a subunit of the H^+^-ATP synthase enzymatic channel). Still, DAPIT cells showed an increased basal respiration and inhibitor-sensitive oxygen consumption of complexes I, II and IV, but decreased activity of H^+^-ATP-synthase. This result is in line with cellular increase in lactate production. Therefore, we suggest that increased maximal respiration is due to increased capacity of complexes I-IV. Since mitochondrial mass was decreased, we conclude that DAPIT positively modulates respiration. In agreement with this hypothesis, we observed increased membrane potential together with citrate synthase activity and VDAC1 expression, an issue suggesting increased availability and use of respiratory chain substrates. As DAPIT cells are glycolytic, these may have altered their catabolic balance in order to fuel the respiration. Accordingly, an accumulation of superoxide production per mitochondria and DAPIT cell was also observed. Interestingly, it was recently reported that intracellular balance of respiratory substrates contribute to the cell decision between differentiation and stemness [54].

Most of the energy needed by human cells is provided by mitochondria in the form of ATP through oxidative phosphorylation. Mitochondrial adenosine triphosphate (ATP) synthesis, while essential to maintain homeostasis, is sensitive to oxidative damages and other cellular injuries [49], and alterations of H^+^-ATP synthase biogenesis increases ROS production while decreasing energy production [55]. ROS damages could disrupt mitochondrial integrity and lead to apoptosis or necrosis, depending on cellular energy status. Regardless of increased mitochondrial respiration and good coupling, the activity of H^+^-ATP-synthase was decreased in DAPIT cells. This decrease could be due to diminished number of H^+^-ATP-synthase complexes in mitochondrial inner membrane, the down-regulation of its enzymatic/hydrolytic activity or both. The decreased H^+^-ATP-synthase activity is well documented in human tumors where the Inhibitory Factor 1 (IF1) of H^+^-ATP-synthase mediates the metabolic shift of cancer cells to aerobic glycolysis with mitochondrial hyperpolarization and subsequent production of superoxide radical [56,57], the mitochondrial characteristics also seen in DAPIT cells. Moreover, the regulated degradation of IF1 controlled energy metabolism during osteogenic differentiation of human mesenchymal stem cells by hindering their self-renewal, but favouring differentiation [58]. These reported studies clarify a mito-cellular mechanism by which the activity of H^+^-ATP-synthase is physiologically regulated in stemness, differentiation and cancer, process where DAPIT over-expression might be involved in. Altogether, these results fit with the idea that DAPIT over-expression accelerates mitochondrial respiration although, or because, inactivating H+-ATP synthase.

Cells can adapt to mitochondrial dysfunctions and energy depletion by regulating mitochondrial biogenesis [49,50]. We observed significant decrease in mtDNA level due to inactivation of mitogenesis in impaired H^+^-ATP-synthase DAPIT cells.

Hif1 induction is reported to shift aerobic cellular metabolism to glycolysis [15–17,59]. Accordingly, translocation of Hif1a to the nucleus was induced in DAPIT cells, and both glucose consumption and lactate production were significantly enhanced. Interestingly, these changes are reminiscent of the Warburg effect observed in many cancers and stem cells.

Hif1α stabilization is also involved in EMT, which is a process of epithelial cells losing cell-cell junctions and baso-apical polarity while acquiring plasticity, mobility, invasive capacity, stem-like characteristics and resistance to apoptosis [60–62]. This cell biology program is active in embryos, fibrosis, wound healing and in promoting metastasis in cancer. In addition to Hif1a, the Wnt/β-catenin pathway signalling also controls EMT upon hypoxic stress in cancer [60,61]. One of the hallmarks of EMT in cancer is the disappearance of E-cadherin from the cellular membrane and its replacement with N-cadherin. Several key transcription factors regulating E-cadherin expression and/or the fate of other epithelial molecules are direct or indirect transcriptional targets of the canonical Wnt pathway [61]. Accordingly, we saw E-cadherin shift to N-cadherin (and regulation of various other proteins) in DAPIT cells and nuclear expression of β-catenin indicating activation of Wnt signalling. All these molecular findings provide evidence that supports the involvement of DAPIT over-expression in altered mitochondrial function in cancer and stemness.

EMT resembling change in DAPIT cells induced transformation of regular cuboidal epithelial-like cells into irregularly sized and shaped cells showing a polygonal, tightly packed, sheet-like appearance with short projections reminiscent of mesenchymal-like cells. However, in contrast to mesenchymal cells, DAPIT cells presented an unexpected decrease in migration capacity. This suggests that some of the defects caused by DAPIT over-expression suppressed the normally improved migratory capacity of mesenchymal-like cells. However, if cell adhesion was unaltered, dissociation from the surface was more frequent.

DAPIT cells grew slower while presenting normal viability. We studied the cell-cycle progression by thymidine synchronization and found that DAPIT cells were arrested in G1. Previously it was shown that the activation of Hif1α (which occurred in DAPIT cells) in embryonic stem cells and colon cancer cells under hypoxia inhibited transcriptional activity of β-catenin resulting in G1 arrest [63,64]. Taken together, the physiological properties of DAPIT cells resemble an EMT-like phenotype with mitochondrial impairment leading to glycolytic metabolism, decreased cell proliferation and migration, and an increase in cell dissociation from the surface, the issues active in varying conditions of cancer and stem cells. Interestingly, searching in the Oncomine cancer genomics database revealed a duplication in *Usmg5* copy number in various cancers (Table 2). This was ranked within 15% of top of duplicated genes in classified brain, pancreas, liver, sarcoma, kidney, lung, gastric, leukemia, breast and ovarian cancers. Despite the link between DAPIT and the tumorigenic capacity has not been sufficiently demonstrated, this result strengthens a correlative involvement of DAPIT in cancer and suggests a possible oncogenic function for it.

In summary, we have characterized the effect of a stable over-expression of DAPIT in a cell culture model; at the level of morphology, molecular biology, metabolic homeostasis and cell behavior. The over-expression of DAPIT in HEK293T cells impaired mitochondria promoting the activation of Hif1α and Wnt/β-catenin signaling, which resulted in a shift of aerobic metabolism to more glycolytic direction and in cell dedifferentiation resembling EMT. We suggest that DAPIT over-expression couples changes in mitochondrial metabolism to physiological and pathophysiological activities at the cellular level, possibly including cancer.

## Supporting Information

S1 Fig Mitochondrial metabolism (activity) at cellular level. Protein levels estimated by Western blot of (A) DAPIT and (B) VDAC. (C) Citrate synthase activity. (D) Protein level of NDUFS3. Inhibitor-sensitive oxygen consumption of (E) complexes I, II, IV and (F) complex V in digitonin-permeabilized and intact cells. Protein level of (G) ATP5a and (H) Sirt3. (I) Basal and maximal respiration. (J) H^+^-ATP synthase activity measured by spectrophotometric analysis. Mitochondrial (K) membrane potential and (L) superoxide levels at cellular level measured by flow cytometry of TMRM (200nM, 30', 37°C) and Mitosox (2,5 μM, 45’, 37°C) stained cells. (M) Protein level of HSP60. Representative immunoblots are shown in Fig 2M. The error bars are S.D. and asterisks indicate: **p<0.01.
(PDF)
