# Correction: DAPIT Over-Expression Modulates Glucose Metabolism and Cell Behaviour in HEK293T Cells

**DOI:** 10.1371/journal.pone.0141036

**Published:** 2015-10-15

**Authors:** Heidi Kontro, Giuseppe Cannino, Pierre Rustin, Eric Dufour, Heikki Kainulainen

There are errors in the “Oncomine data analysis” section of the Materials and Methods. It should read:

Oncomine and CCLE data analysis

We used the Oncomine Cancer Genomics Data Analysis tool [30] and Cancer Cell Line Encyclopedia, CCLE [35] to mine *Usmg5* copy number profiles in a large subset of cancer cell lines [31, 34, 35, 38, 41, 65–69]. In the dataset, the log2 (≥ 0,34) values were analyzed. The number of DNA copies (= 2*(2^y-axis value)) were calculated as advised in Oncomine instructions.

There are errors in the “*Usmg5* copy number in cancers” section of the Results. It should read:

*Usmg5* copy number in cancer cell lines

Since DAPIT over-expression induced EMT and glycolytic switch in HEK293T cells, we tested if DAPIT is over-presented in cancer cell lines. The Oncomine Cancer Genomics database and CCLE revealed a duplication (3–4 copies) of *Usmg5* copy number in a large panel of cell lines (Table 2) Several datasets indicated uniform increase in copy number in various lung (NCI-H1775, NCI-H1993, NCI-H1563, NCI-H1755, VMRC-LCD, SBC-5, NCI-H1703), gastric (HCT116, Hs746T, MKN74, SNU-668), ovarian (OVTOKO, MCAS), liver (SNU-398) and pancreatic (PSN1, PANC-1) cancer cell lines. The copy number was also confirmed in breast (SUM-52PE), endometrial (AN3CA), esophagus (OE33), hematopoietic (MPLM6), kidney (SNU-1272) and lymphoid (Ki-JK) cell lines, being encountered once in the others. These data strongly suggest a role for DAPIT over-expression in cancers.

**Table 2:** Cancer cell lines expressing increased genomic *Usmg5* copy number in Oncomine cancer genomics database and Cancer Cell Line Encyclopedia, CCLE.

Classification	Cancer type	Cell line	DNA copy number	Oncomine dataset/ CCLE
Bone	Osteosarcoma	143B	2,76	CCLE [31]
Brain	Brain gioblastoma	A-172	3,48	Berouchim brain [35]
	Cerebral glioblastoma	LN-18	2,78	Berouchim brain [35]
Breast	Breast adenocarcinoma	MCF7	2,62	Nicolsky Breast [32]
	Breast carcinoma	SUM-52PE	3,60 3,42	Nicolsky Breast [32] Chin breast2 [65]
		CAL-51	3,28	Hu CellLine2 [66]
		MDA-MB-468	2,96	Hu CellLine2 [66]
		CAL-120	2,81	Hu CellLine2 [66]
		Hs 578T	2,74	Hu CellLine2 [66]
		MDA-MB-361	2,72	Hu CellLine2 [66]
		HCC1806	2,62	CCLE [31]
	Ductal breast carcinoma	T-47D	3,18	Nicolsky Breast [32]
		MFM-223	2,85	Nicolsky Breast [32]
	Squamous cell breast carcinoma, Acantholytic variant	HCC1806	2,88	Hu CellLine2 [66]
Central nervous system	Cannabinoid receptor	CB1	2,69	CCLE [31]
	Glioma	KSN60	2,59	CCLE [31]
Endometrium	Endometrial adenocarcinoma	JHUEM2	2,60	CCLE [31]
		AN3CA	2,60 2,59	CCLE [31] Rothenberg CellLine [38]
Esophagus	Eesophageal adenocarcinoma	JHESOAD1	2,61	CCLE [31]
	Barrett's adenocarcinoma	OE33	2,77 2,65	CCLE [31] Wooster CellLine [N/A]
Gallbladder	Biliary tract cancer	SNU478	3,49	CCLE [31]
Gastric	Cecum adenocarcinoma	LS411N	2,70	Lu colorectal [67]
		NCI-H498	2,61	Lu colorectal [67]
		NCI-H747	2,60	Lu colorectal [67]
	Colon adenocarcinoma	SW620	2,88	Lu colorectal [67]
		HCT-15	2,77	Lu colorectal [67]
		LS180	2,72	Lu colorectal [67]
	Colon carcinoma	HCT116	2,94 2,78 2,78 2,77	CCLE [31] Berouchim multicancer [34] Lu colorectal [67] Rothenberg CellLine [38]
	Gastric cancer	Hs 746T	3,54 2,90	Rothenberg CellLine [38] Palanisamy gastric [N/A]
		NCI-N87	3,41	Palanisamy gastric [N/A]
		KATO111	3,34	Palanisamy gastric [N/A]
		YCC-16	3,21	Palanisamy gastric [N/A]
		HUG1N	3,02	CCLE [31]
		YCC-9	3,00	Palanisamy gastric [N/A]
		YCC-6	2,85	Palanisamy gastric [N/A]
		SNU520	2,69	CCLE [31]
	Gastric tubular adenocarcinoma	MKN74	2,74 2,57	CCLE [31] Rothenberg CellLine [38]
	Gastrointestinal stromal tumor	GIST 882X	2,97	Berouchim multicancer [34]
	Signet ring cell gastric adenocarcinoma	SNU-668	2,93 2,80	CCLE [31] Barretina CellLine 2 [31]
Hematopoietic tissue	Acute Myeloid Leukemia	CMK115	2,90	CCLE [31]
		KASUMI1	2,85	CCLE [31]
		CMK	2,67	CCLE [31]
	M2-type of Myeloid Leukemia	KASUMI6	2,78	CCLE [31]
	Blast phase cronic myelogenous leukemia	MOLM6	2,78 2,60	CCLE [31] Barretina CellLine 2 [31]
	Chronic myeloid leukemia	BV173	2,55	CCLE [31]
	Leukemia	NCO2	2,53	CCLE [31]
	Erythroleukemia	TF-1	2,60	Barretina CellLine 2 [31]
Kidney	Clear cell renal carcinoma	SNU-1272	3,13 2,77	CCLE [31] Barretina CellLine 2 [31]
	Renal carcinoma	UOK101	2,87	CCLE [31]
	Human proximal tubular cell line, immortalized	HK2	2,67	CCLE [31]
	Renal adenocarcinoma	ACHN	2,55	CCLE [31]
Liver	Hepatocellular adenocarcinoma	SNU-398	2,84 2,75 2,72 2,59	CCLE [31] Rothenberg CellLine [38] Wooster CellLine [N/A] Barretina CellLine 2 [31]
	Hepatocellular carcinoma	LI7	3,40	CCLE [31]
		C3A	2,62	CCLE [31]
Lung	Lung adenocarcinoma	NCI-HI435	3,15	CCLE [31]
		NCI-H1775	2,73 2,62	CCLE [31] Lu lung [67]
		NCI-H1993	2,79 2,72 2,62	Lu lung [67] Sos CellLine [69] Wooster CellLine [N/A]
		NCI-H1563	2,74 2,68	CCLE [31] Lu lung [67]
		NCI-H1838	2,63	Lu lung [67]
		NCI-H1755	2,86 2,58	Sos CellLine [69] Rothenberg CellLine [38]
		VMRC-LCD	2,67 2,57	CCLE [31] Rothenberg CellLine [38]
		LU65A	2,75	Rothenberg CellLine [38]
	Large cell lung carcinoma	Calu-6	2,59	Lu lung [67]
	Giant cell lung carcinoma	LU65B	3,03	Rothenberg CellLine [38]
	Small cell lung carcinoma	NCI-H510	2,83	CCLE [31]
		DMS53	2,69	Olejniczak CellLine 2 [68]
		SBC-5	2,69 2,64	CCLE [31] Rothenberg CellLine [38]
		DMS114	2,55	CCLE [31]
	Non-small cell lung carcinoma	NCI-H1581	2,67	CCLE [31]
	Squamous cell lung carcinoma	HCC1897	3,53	CCLE [31]
		NCI-HI703	2,63 2,61	CCLE [31] Wooster CellLine [N/A]
	Lung carcinoma	EPLC272H	2,56	CCLE [31]
Lymphoid tissue	Anaplastic large cell lymphoma	Ki-JK	3,21 2,80	CCLE [31] Barretina CellLine 2 [31]
	Non-Hodking B cell Lymphoma	JM1	2,79	CCLE [31]
	Non-Hodking T cell Lymphoma	SR786	2,78	CCLE [31]
		SUDHL1	2,65	CCLE [31]
	Splenic marginal zone B-cell lymphoma	SLVL	2,63	Rothenberg CellLine [38]
Ovarian	Ovarian adenocarcinoma	TOV21G	2,98	CCLE [31]
		OVK18	2,89	CCLE [31]
		CAO V3	2,62	CCLE [31]
	Ovarian clear cell adenocarcinoma	OVTOKO	4,04 3,20	CCLE [31] Barretina CellLine 2 [31]
	Ovarian mucinous custadenocarcinoma	MCAS	3,05 2,92 2,68	CCLE [31] Rothenberg CellLine [38] Barretina CellLine 2 [31]
	Ovarian carcinoma	OVSAHO	2,72	CCLE [31]
	Ovarian carcinoma	OVK12	2,65	Barretina CellLine 2 [31]
	Ovarian granulosa cell tumor	COV434	2,80	CCLE [31]
Pancreas	Ampulla of Vater adenocarcinoma	SNU-478	3,08	Barretina CellLine 2 [31]
	Pancreatic adenocarcinoma	PSN1	2,75 2,61	CCLE [31] Barretina CellLine 2 [31]
		PANC1	2,64 2,53	CCLE [31] Barretina CellLine2 [31]
		PK1	2,53	CCLE [31]
	Panreatic carcinoma	PK45H	2,63	CCLE [31]
		YAPC	2,62	CCLE [31]
Skin	Cutaneous melanoma	A7	2,81	Wooster CellLine [N/A]
	Melanoma	IGR1	2,63	CCLE [31]
	Squamous cell adenocarcinoma	HCC95	2,78	Sos CellLine [69]
		HCC-15	2,59	Sos CellLine [69]
Thyroid	Follicular thyroid carcinoma	ML1	2,53	CCLE [31]

N/A indicating not available

There are errors in Table 2 and in its caption. Please see the corrected Table 2 and its correct caption below.

There are errors in the last sentence in the penultimate paragraph of the Discussion. It should read: Interestingly, searching in the Oncomine cancer genomics database and Cancer Cell Line Encyclopedia, CCLE, revealed a duplication in *Usmg5* copy number in various cancer cell lines (Table 2), highlighting several lung, gastric, ovarian, liver and pancreatic cancer cell lines by supporting fidelity in duplication. The copy number was also confirmed in some breast, endometrial, esophageal, hematopoietic, kidney and lymphoid cell lines. Despite the link between DAPIT and the tumorigenic capacity has not been sufficiently demonstrated, this result strengthens a correlative involvement of DAPIT in cancer and suggests a possible oncogenic function for it.

There are errors in the References. Please view the correct additional references, which are also corrected in Table 3 and the article text described above.

65. Chin SF, Teschendorff AE, Marioni JC, Wang Y, Barbosa-Morais NL, Throne NPet al. High-resolution aCGH and expression profiling identifies a novel genomic subtype of ER negative breast cancer. Genome Biol. 2007;8(10):R215. doi: 10.1186/gb-2007-8-10-r215

66. Hu X, Stern HM, Ge L, O'Brien C, Haydu L, Honchell CD, Haverty PM et al. Genetic alterations and oncogenic pathways associated with breast cancer subtypes. Mol Cancer Res. 2009 Apr;7(4):511–22. doi: 10.1158/1541-7786.MCR-08-0107

67. Lu X, Zhang K, Van Sant C, Coon J, Semizarov D. An algorithm for classifying tumors based on genomic aberrations and selecting representative tumor models. BMC Med Genomics. 2010 Jun 22;3:23. doi: 10.1186/1755-8794-3-23

68.Olejniczak ET, Van Sant C, Anderson MG, Wang G, Tahir SK, Sauter G et al. Integrative genomic analysis of small-cell lung carcinoma reveals correlates of sensitivity to bcl-2 antagonists and uncovers novel chromosomal gains. Mol Cancer Res. 2007 Apr;5(4):331–9. Doi: 10.1158/1541-7786

69. Sos ML, Michel K, Zander T, Weiss J, Frommolt P, Peifer M et al. Predicting drug susceptibility of non-small cell lung cancers based on genetic lesions. J Clin Invest. 2009; Jun;119(6):1727–40. doi: 10.1172/JCI37127
