# Correction: DAPIT Over-Expression Modulates Glucose Metabolism and Cell Behaviour in HEK293T Cells

**DOI:** 10.1371/journal.pone.0143268

**Published:** 2015-11-12

**Authors:** Heidi Kontro, Giuseppe Cannino, Pierre Rustin, Eric Dufour, Heikki Kainulainen

There are errors in the References. Please view the correct additional references here.

31. Barretina J, Caponigro G, Stransky N, Venkatesan K, Margolin AA, Kim S, et al. The cancer cell line encyclopedia enables predictive modelling of anticancer drug sensitivity. Nature. 2012;483(7391):603–607. doi: 10.1038/nature11003

32. Nikolsky Y, Sviridov E, Yao J, Dosymbekov D, Ustyansky V, Kaznacheev V, et al. Genome-wide functional synergy between amplified and mutated genes in human breast cancer. Cancer Res. 2008;68(22):9532–9540. doi: 10.1158/0008-5472.CAN-08-308

34. Beroukhim R, Mermel CH, Porter D, Wei G, Raychaudhuri S, Donovan J, et al. The landscape of somatic copy-number alteration across human cancers. Nature. 2010;463(7283):899–905. doi:10.1038/nature08822

35. Beroukhim R, Getz G, Nghiemphu L, Barretina J, Hsueh T, Linhart D, et al. Assessing the significance of chromosomal aberrations in cancer: Methodology and application to glioma. Proc Natl Acad Sci U S A. 2007;104(50):20007–20012. doi: 10.1073/pnas.0710052104

38. Rothenberg SM, Mohapatra G, Rivera MN, Winokur D, Greninger P, Nitta M, et al. A genome wide screen for microdeletions reveals disruption of polarity complex genes in diverse human cancers. Cancer Res. 2010;70(6):2158–2164. doi: 10.1158/0008-5472.CAN-09-3458
