# Supplementary figures and images for: DAPIT Over-Expression Modulates Glucose Metabolism and Cell Behaviour in HEK293T Cells
Source: PLoS One. 2015 Jul 10;10(7):e0131990. doi: 10.1371/journal.pone.0131990 (PMC4498893; doi:10.1371/journal.pone.0131990)

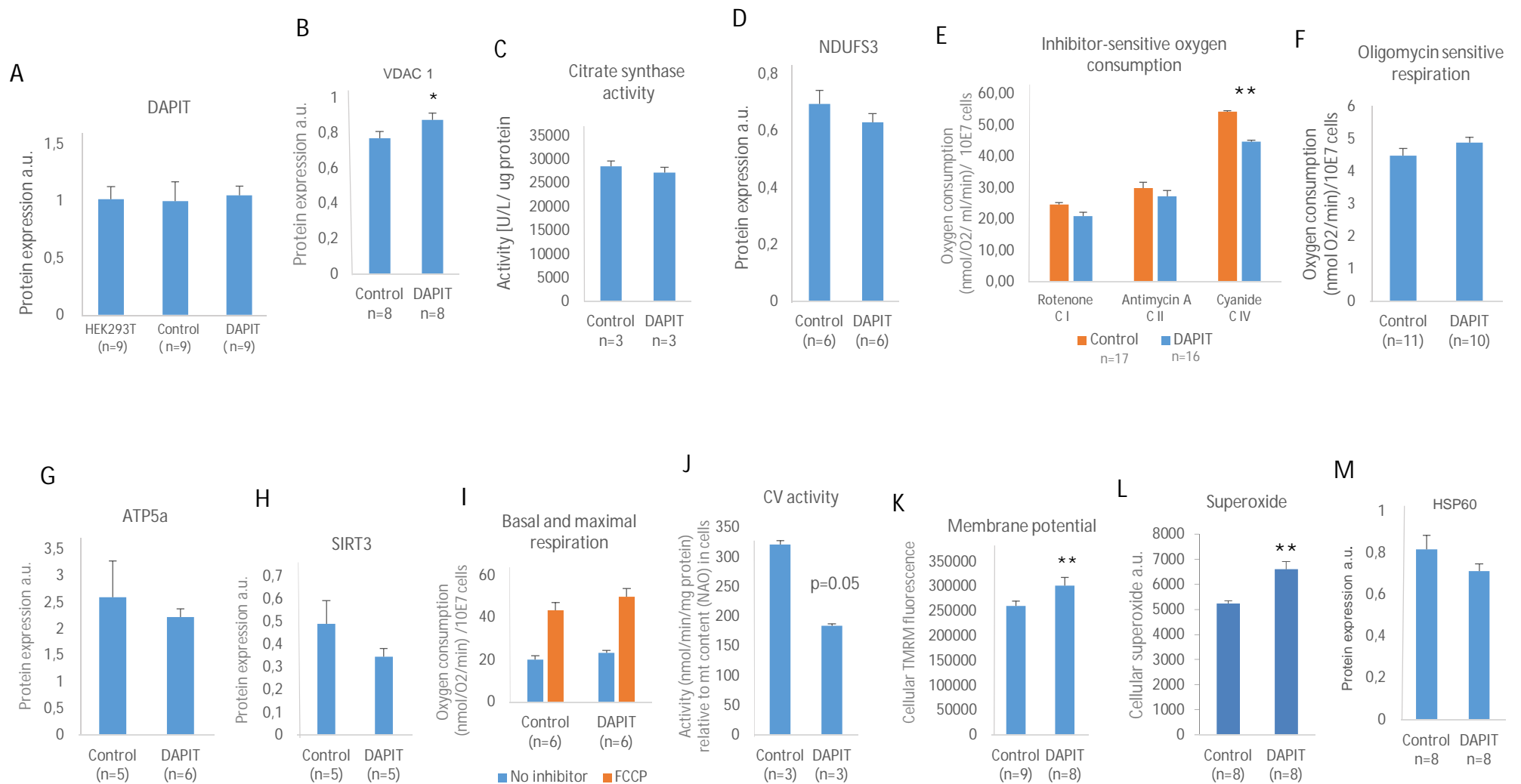

Supplement: S1 Fig — Protein levels estimated by Western blot of (A) DAPIT and (B) VDAC. (C) Citrate synthase activity. (D) Protein level of NDUFS3. Inhibitor-sensitive oxygen consumption of (E) complexes I, II, IV and (F) complex V in digitonin-permeabilized and intact cells. Protein level of (G) ATP5a and (H) Sirt3. (I) Basal and maximal respiration. (J) H+-ATP synthase activity measured by spectrophotometric analysis. Mitochondrial (K) membrane potential and (L) superoxide levels at cellular level measured by flow cytometry of TMRM (200nM, 30', 37°C) and Mitosox (2,5 μM, 45’, 37°C) stained cells. (M) Protein level of HSP60. Representative immunoblots are shown in Fig 2M. The error bars are S.D. and asterisks indicate: **p<0.01. (PDF) [file pone.0131990.s001.pdf]
